# Radiographic analysis of subclinical appearances of the hip joint among patients with labral tears

**DOI:** 10.1186/s13018-019-1435-z

**Published:** 2019-11-14

**Authors:** Hisaki Aiba, Nobuyuki Watanabe, Muneyoshi Fukuoka, Ikuo Wada, Hideki Murakami

**Affiliations:** 1 Department of Orthopaedic Surgery, Tosei General Hospital, 160 Nishioiwake-cho, Seto, Aichi 489-8642 Japan; 2 Department of Orthopaedic Surgery, Nagoya City University Graduate School of Medical Sciences, 1, Kawasumi, Mizuho-cho, Mizuho-ku Nagoya, Aichi 467-8601 Japan; 3 Department of Rehabilitation Medicine, Nagoya City University Graduate School of Medical Sciences, 1, Kawasumi, Mizuho-cho, Mizuho-ku Nagoya, Aichi 467-8601 Japan

**Keywords:** Hip arthroscopy, Labral tear, Multiplanar reconstruction CT, Femoral acetabular impingement

## Abstract

**Objective:**

Labral tears can be complicated by hip diseases, including osteoarthritis or femoral acetabular impingement. To accurately plan hip arthroscopy or subsequent conversion to total hip arthroplasty, the presence of bony abnormalities in the hip joint must be evaluated. This study aimed to elucidate the utility of multiplanar reconstruction computed tomography (mCT) for the detection of subclinical coincidence of osteoarthritis or femoral acetabular impingement with a labrum tear.

**Materials and methods:**

We retrospectively analysed 34 patients (36 hips) with labrum tears without apparent osteoarthritis or hip dysplasia from 2012 to 2015. The joint spaces were calculated using radiographs or mCT, and the detection rates of degenerative cyst and herniation pit were compared.

**Results:**

Narrow joint spaces (< 2 mm) were more clearly detected in mCT (*p* < 0.05, chi-square analysis) than in radiographs. The detection rate of cysts in the acetabulum was 8.3% using radiographs and 36.1% using mCT (*p* < 0.001, chi-square analysis). Additionally, the detection of herniation pit was 8.3% and 25.0% using radiographs and mCT, respectively (*p* = 0.053, chi-square analysis).

**Conclusion:**

We performed the radiographic analysis of patients with labral tears using radiographs and mCT. The mCT allowed for fine detection of narrow joint spaces and subtle subclinical appearances. The results of this study may provide surgeons with more appropriate strategies for the treatment of labral tears.

## Introduction

The acetabular labrum is an essential structure for hip joint stability [1]. The loss of integrity of the structure induces micro-instability, subluxation, or hip pain [2]. The injury can be preceded by osteoarthritis (OA), hip dysplasia, or femoral acetabular impingement (FAI) [3, 4]. With progress in minimally invasive surgery, arthroscopic surgery has been indicated for the treatment of labral tear. However, the incidence of OA with labral tear adversely affects the outcomes following arthroplasty [5]. To evaluate the osteoarthritic changes in the joint, the “2-mm rule” was proposed by Philippon et al. for the prediction of poor outcomes after hip arthroscopic surgery [6]. The joint space is measured at the hip joint, and the 2-mm cut-off value predicts the necessity to perform total hip arthroplasty (THA) after arthroscopic surgery [6]. Originally, the 2-mm rule was evaluated using radiographs. However, in clinical settings, diagnostic imaging is often performed by other multimodal approaches. Computed tomography (CT) is considered more sensitive than radiographs for the precise detection of bone cysts, including herniation pits which indicate cam-type FAI [7], and subchondral cysts of the acetabulum, which indicate OA changes. Further, a multiplanar reconstruction CT (mCT) image can precisely measure the joint space and reduce intra-observer error. This study aimed to elucidate the utility of mCT for the detection of subclinical coincidence of OA or FAI with a labrum tear.

## Methods

From 2012 to 2015, 51 patients (53 hip joints) were diagnosed with labral tears using radial magnetic resonance imaging (MRI) with contrast agent. Seven patients with apparent OA in the hip joint (Tonnis grade ≥ 2), acetabular dysplasia (centre edge angle < 25), or coxa plana following Perthes disease were excluded. Ten patients were excluded from this study owing to indistinct images. Finally, 34 patients (36 hip joints) were assigned to the cohort in this study. The patients’ characteristics are shown in Table 1. The cohort was observed for 419 days ± 207 (mean ± standard deviation).

**Table 1:** Clinical and treatment characteristics of patients

	Characteristics	Number	Percentage
Sex	Male	10	27.8
	Female	26	72.2
Position	Left	23	63.9
	Right	13	36.1
Czerny classification	1a-b	9	25.0
	2a-b	15	41.7
	3a-b	12	33.3
Treatment	Conservative	17	47.2
	Surgery	19	52.8
Age at diagnosis, years	41.6 ± 10.7 (mean ± standard deviation)		
JOA score at diagnosis	72.8 ± 18.2 (mean ± standard deviation)		
Harris Hip Score at diagnosis	76.9 ± 14.2 (mean ± standard deviation)		

### Measurement of joint space in the hip joint

#### Radiographs

**Fig. 1:**
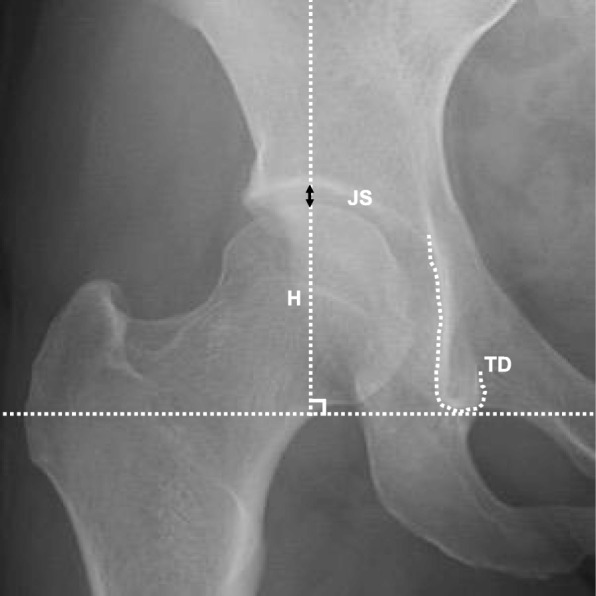
Method of joint space measurement by radiographs. Measurement of the joint space (JS) in the anteroposterior view of the radiographs. JS measured between the femur and acetabulum on the perpendicular line passing the femoral head (H) from the connecting line of both teardrops (TD)

Antero-posterior pelvic (AP) view radiographs were obtained from the patients, who were in supine positions with the legs internally rotated 15° [8]. We defined the length of hip joint space as the length between the cortical bone of the acetabulum and the cortical bone of the femoral head on the perpendicular line between the centre of the femoral head (Fig. 1). Measurements were taken three times, and the means were calculated.

#### CT

**Fig. 2:**
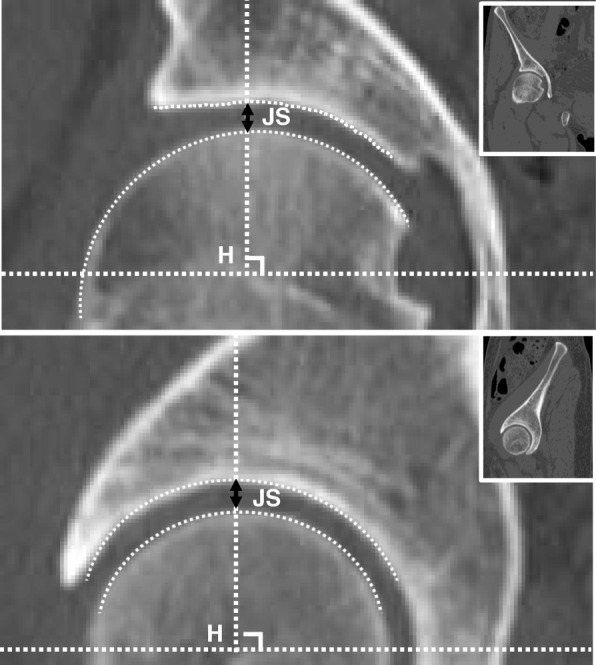
Method of joint space measurement by multiplanar reconstruction computed tomography (CT). The calculations of the joint space (JS) were performed between the surface of the cortical bone of the acetabulum and that of the femoral head on the perpendicular line from the centre of the femoral head (H). The appropriate point of sagittal and coronal views was determined at the slice where the size of the femoral head was most prominent. The upper row indicates the coronal view of mCT, and the lower one indicates the sagittal view. The wickets indicate a wider view of mCTs

The calculations of the joint space were performed similarly between the cortical bone of the acetabulum and cortical bone of the femoral head on the perpendicular line from the centre of the femoral head. From the mCT, the sagittal and coronal views were determined in the slice in which the femoral head appeared maximal in size (Fig. 2). The device and conditions of mCT were Aquilion PRIME (Canon, Tokyo) at 0.5 s/rotation (helical pitch = 27.0), respectively.

### Detection of degenerative cysts in the hip joint

We also analysed the cystic changes in the acetabulum owing to OA and herniation pit, which is defined as a cystic lesion underneath the anterior cortex at the antero-superior femoral head-neck junction with clear demarcation and diameter of > 3 mm using radiographs and mCT (coronal view) [9, 10].

### Indication of operation

First, all patients underwent conservative therapy with stretching of the flexor muscles of the hip joint and core muscle training of the trunk and pelvis for at least 6 months. If the symptoms did not improve enough to allow for participation in desired sports activities and if there were still barriers to activities of daily life, school attendance, or employment, the labrum was repaired with arthroscopic assistance. The procedures of the surgery were performed in accordance with those reported by Kelly et al. [11]. Furthermore, osteoplasty of the femoral head was added for cases of cam-type FAI.

### Physical therapy

Physical therapy after surgery was divided into four phases.

- Phase 1 (0–4 weeks): in this period, the aim was to protect the sutured labrum tear and achieve joint movement. Weight-bearing was restricted for 2 weeks after surgery and gradually increased from 1/3 partial weight-bearing with 1/3 augmentations per week. With respect to passive training for the range of motion, 60° flexion and 30° abduction of the hip joint were permitted during the first week postoperatively. For the second week after surgery, 90° flexion and 30° external rotation of the hip joint were permitted, with no restriction on abduction and internal rotation. Three weeks after surgery, 120° flexion was permitted, and abduction and external and internal rotation were not restricted. Phillipon hip brace (BREG, Carlsbad, CA) was used at night and at rest until 2 weeks after surgery. Moreover, the physical therapist massaged the soft tissues and aided in the mobilisation of the operative scar tissue.
- Phase 2 (4–8 weeks): in this period, the aim was stabilisation of movement and achievement of weight-bearing. Active flexion, abduction, and external rotation were performed, including the use of the exercise bike, underwater walking, performing plank exercise, and training of the quadriceps and gluteus medius.
- Phase 3 (8–12 weeks): in this period, the aim was to augment the muscle strength. Therefore, regular exercises were combined with closed kinetic chain hip strengthening, one-third knee bends, slide-to-slide lateral movement, and forward box lunge.
- Phase 4 (after 12 weeks): in this period, the aim was to return to sports activity. Sports-specific training was performed, and the functionality for the return to sports activity was assessed.

### Functional evaluation

Patient function at diagnosis and at the last visit were evaluated by the Japanese Orthopaedic Association (JOA) Hip Score [12] and Harris Hip Score [13].

### Statistical analysis

A *p* value < 0.05 was considered statistically significant. All values were presented either as mean ± standard deviation or median with range, depending on the distribution. All statistical analyses were conducted using SPSS version 24 (IBM, Chicago, IL). Radiographical images were evaluated independently and cross-checked by two orthopaedic surgeons (H.A. and N.W.), who were blinded to all other patient information. The kappa value was then calculated to evaluate the intra-observer (N.W., over a 1-month interval) and inter-observer (H.A. and N.W.) error. The comparisons of numeric values were performed by paired *t* test or Mann-Whitney *U* test based on the distributions.

## Results

### Measurement of the joint space from the AP radiographs and the coronal or sagittal CT

The mean length of the joint space was 4.3 mm on the radiographs, 3.2 mm on the coronal CT, and 3.2 mm on the sagittal CT (significant differences were found between radiographs and mCTs [*p* < 0.05, paired *t* test]). Moreover, narrow joint spaces (< 2 mm) were sharply detected in mCT (Tables 2 and 3, *p* = 0.012 [coronal CT], not significant [sagittal CT]) than in radiographs. For the detection of narrow joint space, the intra-observer errors (N.W., over a 1-month interval) were 0.78, 0.61, and 0.71 (radiographs, coronal CT, and sagittal CT, respectively) and the inter-observer errors (N.W. and H.A.) were 0.65, 0.50, and 0.61 (radiographs, coronal CT, and sagittal CT, respectively).

**Table 2:** Analysis of joint space with different modalities (coronal CT)

Detection of narrow joint space (coronal CT)				
	Joint space calculated by multiplanar computed tomography (coronal)			
Joint space calculated by radiographs		< 2 mm	≥ 2 mm	Total
	< 2 mm	1	0	1
	≥ 2 mm	4	31	35
	Total	5	31	36

**Table 3:** Analysis of joint space with different modalities (sagittal CT)

Detection of narrow joint space (sagittal CT)				
	Joint space calculated by multiplanar computed tomography (sagittal)			
Joint space calculated by radiographs		< 2 mm	≥ 2 mm	Total
	< 2 mm	1	0	1
	≥ 2 mm	7	28	35
	Total	8	28	36

**Table 4:** Subclinical appearance of the hip with labrum tear (presence of cysts by CT)

Detection of the degenerative cyst in the acetabulum				
	Presence of cysts by CT (length of cyst; mm)			
Presence of cysts by radiographs		Positive	Negative	Total
	Positive	3 (11.1 ± 3.6)*	0	3
	Negative	10 (3.5 ± 1.5)	23	33
	Total	13	23	36

*p < 0.001; comparison between the length of cysts (mean *±* standard deviation) based on different modalities (Mann-Whitney *U* test)

### Validation of degenerative cysts in the acetabulum

We analysed the cysts in the acetabulum using radiographs and mCT images. The detection rate was 8.3% (3/36) in the radiographs and 36.1% (13/36) in mCT. Based on the chi-square analysis, there was a statistical difference in the detection rate in both modalities (*p* < 0.001). The average length of the cyst in the radiograph-negative case (*n* = 10) was 3.5 mm (SD 1.5 mm), indicating the presence of the detection limit of the radiographs (Table 4). For the detection of degenerative cysts, the intra-observer errors (N.W., over a 1-month interval) were 0.65 and 0.68 (radiographs and mCT, respectively) and the inter-observer errors (N.W. and H.A.) were 0.53 and 0.46 (radiographs and mCT, respectively).

### Validation of the herniation pit in the femoral head

We analysed the cysts in the acetabulum using radiographs and mCT images. The detection rate was 8.3% (3/36) with radiographs and 25.0% (8/36) with mCT (Table 5). From a chi-square analysis, no statistical difference was found in the detection rate of these modalities (*p* = 0.053). For detection of the herniation pit, the intra-observer errors (N.W., over a 1-month interval) were 0.24 and 0.63 (radiographs and mCT, respectively) and inter-observer errors (N.W. and H.A.) were 0.36 and 0.52 (radiographs and mCT, respectively).

**Table 5:** Subclinical appearance of the hip with labrum tear (presence of herniation pit cysts by CT)

Detection of the herniation pit				
	Presence of herniation pit cysts by CT			
Presence of herniation pit by radiographs		Positive	Negative	Total
	Positive	2	1	3
	Negative	6	27	33
	Total	8	28	36

### Clinical outcomes of the cohort

In this cohort, regardless of operation or conservative treatment, almost all patients had improved JOA and Harris Hip Scores. Moreover, THA after hip arthroscopy was only performed in one patient, owing to fracture and dislocations of the hip joint secondary to a traffic accident. However, because almost all patients with or without surgery showed improved functionality, the effect of the length of joint space and the detection of OA change and herniation pit were not strongly related to the poor outcomes of the patients (Additional file 1: Table S1).

## Discussion

We investigated the subclinical deformities in patients with an acetabular labral tear. First, we validated the application of mCT for measurement of the joint space. Moreover, to assist in the diagnosis of OA or cam-type FAI, we compared the cystic changes in the acetabulum and femoral head (herniation pit) using radiographs and mCT. The lengths of the joint spaces measured by mCT tended to be smaller than those calculated by radiographs. This might be due to the sensitivity of mCT, allowing measurements at any point. Specifically, mCT can measure where the size of the femoral head is the largest and the joint space smallest. Additionally, as was expected, the cyst in the acetabulum and the herniation pits were more sharply detected using mCT. These results provided thorough subclinical details of the concurrent OA or FAI and more prudent preoperative planning before hip arthroscopy.

It is difficult to clearly distinguish between these concurrent hip diseases. The cohort of asymptomatic men (*n* = 244) elucidated that the cam-type deformity was related to the herniation pit, labrum tear, and decrease in the cartilage thickness [14]. A cross-sectional Japanese population study revealed that the rate of coexistence of acetabular dysplasia was 23.4% in patients with FAI [9]. On the contrary, owing to the mechanical stress to the labrum, the dysplastic hip is at risk for abnormal thickening of the labrum [15]. Thus, subclinical deformities of the hip should be assessed using multiple diagnostic techniques prior to making treatment decisions [16].

Joint space is one of the factors that affects the outcome after surgery. Philippon et al. reported that the conversion rate to THA after hip arthroscopy was 39 times higher in patients with < 2-mm joint space than those with ≥ 2 mm [6]. McCormick et al. also stated the importance of the 2-mm rule in that the conversion rate to THA after hip surgery was higher in patients with < 2-mm joint space (86% versus 16%) [17]. However, in this study, the 2-mm rule was not related to the symptoms at diagnosis, clinical outcomes, or conversion rate. This is partially because we excluded patients with apparent OA change (Tonnis grade ≥ 2), resulting in a lower conversion rate to THA. However, we should be careful when considering the predisposing factors for poor prognosis prior to treatment.

In this study, we did not prove the relation between patient-reported outcomes and prevalence of subclinical hip deformities. Generally, with the narrowing of the joint space and the progression of OA change, the range of motion and hip pain become worse [18]. In addition, according to the Copenhagen City Heart Study, a joint space < 2-mm was strongly related to coxalgia (odds 3.2–3.3) and more severe OA change correlated with coxalgia (odds 17.4) [19]. However, moderate changes were only mildly related to pain (odds 1.4), especially in the younger population (under 50 years). This result was similar to that of the current study, partially because the present cohort included mainly a younger population (mean age 41.6 years). Thus, it was postulated that subclinical findings in the hip joint did not apparently affect patient-reported outcomes; however, careful attention must be paid to subtle changes in the hip joint, paying attention specifically to long-term effects.

This study has limitations that might have affected the results. First, selection bias is present. We included patients with labral tears observed in radial MRI with a contrast agent, and patients with apparent OA were excluded from this study. Further, the population who responded well to conservative therapy was exclusively selected. Second, the study analysis was dependent on the resolution of the software and hardware. This might relate to measurement error and influence external validity. Third, the number of the population was relatively smaller, and the adequacy of the follow-up periods was still unclear. Despite these limitations, the detection of narrow joint space and degenerative cysts should be fully assessed with mCT to differentiate concomitant hip diseases.

## Conclusion

We performed radiographic analysis of patients with labral tears using radiographs and mCT. Using mCT, narrow joint spaces and subtle subclinical appearances were finely detected. These findings may provide surgeons with more appropriate strategies for the treatment of labral tears.

## Supplementary information

**Additional file 1.** **Table S1.** Clinical outcomes of the patients with relations to the predisposing factors.

## Data Availability

The datasets used and analysed during the current study are available from the corresponding author on reasonable request.

## Abbreviations

AP: Antero-posterior pelvic
FAI: Femoral acetabular impingement
JOA: Japanese Orthopaedic Association
mCT: Multiplanar reconstruction computed tomography
MRI: Magnetic resonance imaging
OA: Osteoarthritis
THA: Total hip arthroplasty
