# Supplementary material for: Radiographic analysis of subclinical appearances of the hip joint among patients with labral tears
Source: J Orthop Surg Res. 2019 Nov 14;14:369. doi: 10.1186/s13018-019-1435-z (PMC6854768; doi:10.1186/s13018-019-1435-z)
Supplement: Supplementary file 1 — Additional file 1. Table S1. Clinical outcomes of the patients with relations to the predisposing factors. [file 13018_2019_1435_MOESM1_ESM.docx]

Additional file 1: **Table S1.** Clinical outcomes of the patients with relations to the predisposing factors.

|  | Characteristics | Number | Score at diagnosis | | Score at final follow-up | |
| --- | --- | --- | --- | --- | --- | --- |
| Treatment | Conservative  Operation | 17  19 | **83.6 ± 11.0 (JOA)  63.0 ± 18.0 (JOA) | **83.6 ± 11.0 (HHS)  70.9 ± 14.0 (HHS) | 93.6 ± 5.6 (JOA)  93.9 ± 5.0 (JOA) | *93.6 ± 5.7 (HHS)  96.9 ± 3.1 (HHS) |
| Joint space  (by X-ray) | <2 mm  ≥2 mm | 1  35 | 92.0 (JOA)  72.2 ± 18.2 (JOA) | 92.0 (HHS)  76.5 ± 14.2 (HHS) | 91.0 (JOA)  93.9 ± 5.3 (JOA) | 91.0 (HHS)  95.5 ± 4.7 (HHS) |
| Joint space  (by coronal CT) | <2 mm  ≥2 mm | 5  31 | 75.6 ± 14.4 (JOA)  72.3 ± 18.9 (JOA) | 75.0 ± 14.8 (HHS)  77.2 ± 14.3 (HHS) | 90.6 ± 6.2 (JOA)  94.3 ± 5.0 (JOA) | 93.8 ± 7.1 (HHS)  95.6 ± 4.3 (HHS) |
| Joint space  (by sagittal CT) | <2 mm  ≥2 mm | 6  30 | 75.5 ± 13.2 (JOA)  72.2 ± 19.1 (JOA) | 75.0 ± 13.5 (HHS)  77.3 ± 14.5 (HHS) | 92.8 ± 6.8 (JOA)  94.0 ± 5.0 (JOA) | 95.7 ± 6.6 (HHS)  95.3 ± 4.4 (HHS) |
| Presence of cyst | Yes  No | 13  23 | 75.6 ± 16.4 (JOA)  71.1 ± 19.3 (JOA) | 77.9 ± 13.2 (HHS)  76.4 ± 15.0 (HHS) | 93.2 ± 4.8 (JOA)  94.1 ± 5.5 (JOA) | 93.5 ± 5.2 (HHS)  96.4 ± 4.2 (HHS) |
| Presence of herniation pit | Yes  No | 8  28 | 78.1 ± 20.4 (JOA)  71.4 ± 17.8 (JOA) | 81.7 ± 15.7 (HHS)  75.8 ± 13.9 (HHS) | 92.7 ± 6.0 (JOA)  94.0 ± 5.1 (JOA) | 92.9 ± 6.1 (HHS)  96.0 ± 4.2 (HHS) |

**, p<0.01; *, p<0.05; comparison between characteristics at the same periods (paired *t* test)

JOA; Japan Orthopaedic Association, HHS; Harris Hip Score.
